# Advanced Computational Approaches for Medical Genetics and Genomics

**DOI:** 10.1155/2015/705469

**Published:** 2015-07-30

**Authors:** Zhi Wei, Xiao Chang, Junwen Wang

**Affiliations:** ^1^Department of Computer Science, New Jersey Institute of Technology, Newark, NJ 07102, USA; ^2^The Center for Applied Genomics, Children's Hospital of Philadelphia, Philadelphia, PA 19104, USA; ^3^Centre for Genomic Sciences and Department of Biochemistry, LKS Faculty of Medicine, The University of Hong Kong, Hong Kong

Remarkable advances have been made in genetics and genomics over the last decade due to the rapid technology innovation in microarray and sequencing. Thanks to biomedical discoveries, it is feasible now to improve the diagnosis and treatment by genetic and genomic tests, for paving the way for an era of personalized/precision medicine in health care. However, it remains challenging to identify causal mutations from massive amounts of genomic data. There is an unprecedented demand for novel computational methods and analytical strategies to improve the accuracy of variants identification and the power of association tests. This special issue aims to publish applications of innovative analysis pipelines and algorithms to find the better solutions of complex genetic and genomic problems in a time efficient manner. We look for original research findings and practical applications that contribute to the diagnosis and management of human disorders.

In this special issue, we selected ten papers from dozens of submissions after in-depth review. We summarize their key contributions and findings as follows.

In the field of* medical genetics*, we collected three research papers and one review paper. S. Perez-Alvarez et al. developed a statistical algorithm (FARMS) for variable selection aiming to identify variables with the optimal predication performance for a specific outcome. The authors further applied FARMS to a high-dimensional dataset of over 800 individuals and showed that the proposed method is more efficient than other approaches such as regression based method. Y. Wang et al., in another methodology paper, proposed a novel method (LRSDec) to identify gene modules and genetic interactions between them. It is based on regularized low-rank approximation and enjoys nearly optimal error bounds. We expect its wide applications in the field of genetic interaction data analysis, image processing, and so on. Using a statistical genomic approach T. Du et al. reported the discovery of the association between FSHR polymorphisms and polycystic ovary morphology in women with polycystic ovary syndrome. In a review article, R. de Vlaming and P. J. F. Groenen surveyed the use of ridge regression for prediction in quantitative genetics by genotyping data. They also performed a suite of simulations to estimate the effect of sample size, the number of SNPs, and trait heritability on the accuracy of the results.

In the field of* medical genomics*, we collected six research articles. Four of them employed network-based computational strategies to investigate the molecular mechanisms of complex diseases including cancer, obesity, and Type 2 Diabetes (T2D). By computing coexpression gene pairs in two types of lung cancers and the normal lung tissues, F. Long et al. identified molecular biomarkers that distinguish small-cell lung cancer and non-small-cell lung cancer. In another article, Q. Zou et al. proposed a network-based method to predict the association between microRNAs and diseases and further developed it into a web server (http://datamining.xmu.edu.cn/~jinjinli/MircoDAP.html.) for use by the community. In “Network-Based Association Study of Obesity and Type 2 Diabetes with Gene Expression Profiles,” S. Zhang et al. integrated multiple omics data of obesity and T2D to construct a comprehensive biological network. Their novel strategy revealed the pathways associated with both obesity and T2D. Another article by S. Park et al. explored the impact of inflammatory responses to the risk of colorectal cancer and Alzheimer's disease in the view of systems biology. Y. Sun and Q. Liu presented a computational framework for deciphering the correlation between breast tumor samples and cell lines. They proposed to integrate both copy-number changes and gene expression profiles. Their investigation can serve as a useful guide to bridge the gap between cell lines and tumors and help to select the most suitable cell line models for personalized cancer studies. The article by T. Liu et al. conducted a gene coexpression and evolutionary conservation analysis of the human preimplantation embryos. Their study demonstrated the patterns of evolutionary constraints that were imposed on different stages of human preimplantation embryos.

We believe the publications in this collection will attract and benefit readers from multiple disciplines, not only bioinformaticians and statistical geneticists for methodology development, but also researchers and clinicians in using the novel tools for their science discovery.

